# Correlation Analysis of Hemoglobin-to-Red Blood Cell Distribution Width Ratio and Frailty in Elderly Patients With Coronary Heart Disease

**DOI:** 10.3389/fcvm.2021.728800

**Published:** 2021-08-27

**Authors:** Jiling Qu, Ting Zhou, Mengxin Xue, Huiping Sun, Yijing Shen, Yuhui Chen, Lei Tang, Lin Qian, Jiachun You, Ruohan Yang, Yongbing Liu

**Affiliations:** School of Nursing, Yangzhou University, Yangzhou, China

**Keywords:** hemoglobin-to-red blood cell distribution width ratio, frailty, inpatients, coronary heart disease, older adults, association

## Abstract

**Background:** Coronary heart disease (CHD) is a common chronic disease in the elderly. Frailty can accelerate the development of CHD and lead to adverse health outcomes. Risk prediction and decision-making for frailty are crucial. The peripheral hemoglobin-to-red blood cell distribution width ratio (HRR) is a novel biomarker of inflammation. Our purpose was to explore the correlation between HRR and frailty in elderly patients with CHD.

**Methods:** This cross-sectional study evaluated 245 Chinese hospitalized patients with CHD. Blood parameters measured upon admission were obtained *via* the hospital electronic information medical record system. The Fried Frailty Phenotype Scale was used to evaluate the frailty status of the participants. The Receiver operating characteristic curve was used to determine the optimal cut-off values of HRR. We used univariate analysis to examine the potential factors affecting frailty. Kendall's tau-b grade correlation was used to analyze the correlation between HRR and frailty. The ordered logistic regression model was used to analyze the relationship between HRR and frailty.

**Results:** A total of 233 elderly patients with CHD were included in our study. Among the patients, 33.48% (78) were in a state of frailty. The optimal cut-off values of HRR was 9.76. The area under the curve (AUC) for HRR in the frailty patients was 0.652, exceed Hb (AUC = 0.618) and RDW (AUC = 0.650). Kendall's tau-b grade correlation analysis showed that HRR (K = −0.296, *P* < 0.001) was negatively correlated with frailty. The ordered logistic regression analysis determined that lower HRR was associated with frailty (*P* < 0.05) after adjusted for age, body mass index, number of drugs, comorbidity index, heart failure, red blood cells, albumin, total cholesterol, triglyceride, high density lipoprotein cholesterol, and low density lipoprotein cholesterol.

**Conclusion:** Lower HRR is an independent risk factor for frailty in elderly hospitalized patients with CHD. HRR was a more powerful prognostic indicator for frailty than either Hb or RDW alone. Clinicians should focus on timely identification of the risk of frailty in order to improve patient quality of life and to reduce the risk of complications.

## Introduction

Frailty is a geriatric syndrome caused by a cumulative decline in multiple physiological systems, leading to a decrease in the reserve and resistance to stressors (1). It is associated with longer hospital stays, increased medical costs, and increased mortality (2–4). Coronary heart disease (CHD) is a common chronic disease in the elderly. Studies have shown that frailty is an independent risk factor for the accelerated development of age-related diseases in patients with CHD (5). Yoshioka et al. (6) have found that intermediate mortality in patients with ST-segment myocardial infarction is associated with frailty. Frailty can be reversed with intervention, especially in the early stages (7). A recent review and meta-analysis found that only 3% of frail older adults can spontaneously return to a robust state (8). Therefore, frailty risk prediction and decision-making has an important role in patients with CHD.

It is well-known that a complete blood count (CBC) is part of a routine checkup for patients upon admission to the hospital. Several studies have shown that certain markers in CBC are strongly associated with frailty (9–12). Bodolea et al. (9) found a correlation between platelet count (PLT) and frailty in patients with cardiovascular disease. The neutrophil-to-lymphocyte ratio (NLR) is another example that has been widely studied by researchers in recent years. Hou et al. (10) showed that NLR is an independent risk factor for frailty in elderly patients with CHD. Nishijima et al. (13) found a significant positive association between frailty and NLR in older cancer patients.

Hemoglobin (Hb) is an important parameter of CBC, to some extent reflecting the degree of anemia in patients. A prior meta-analysis (11) identified five biomarkers (C-reactive protein (CRP), Hb, albumin, 25-hydroxyvitamin D (25OHD), and free testosterone) that are strongly associated with frailty. In the findings from the Singapore Longitudinal Study of Aging, Hb (g/dL; adjusted for sex, age, and education) was found to be a debilitating risk factor (14).

Red cell distribution width (RDW) is another important parameter of CBC, which has been used to measure the variability of red blood cell (RBC) count to diagnose different types of anemia and for differential diagnosis. In recent years, it has been found that the changes in RDW are closely related to the inflammatory response of the host system (15, 16). In subjects with joint pain, RDW interpretation, like CRP, is a useful tool in clinical practice to distinguish between inflammatory and non-inflammatory joint diseases (17). Hou et al. (10) found that RDW is an independent risk factor for frailty in elderly patients with CHD.

Since RDW is affected by complex clinical conditions, the effect of RDW on frailty is not only related to its inflammatory response, but also to overall sub-optimal health, indicating a decrease in the ability of the system to repair recovery and oxygen. Although previous studies have shown encouraging results, we believe that RDW without other indicators by itself may not be able to reflect systemic inflammatory status and provide definitive predictive information. The peripheral hemoglobin-to-red blood cell distribution width ratio (Hb/RDW, HRR) is a novel marker of inflammation. It was first proposed by Sun et al. (18) and verified in patients with esophageal squamous cell carcinoma. They found that HRR was a more powerful prognostic indicator than either Hb or RDW alone. This is thought to be because HRR combines prognostic information from Hb and RDW and provides more information than a single variable (18).

In conclusion, it is reasonable to believe that HRR is a more stable predictor of frailty. To our knowledge, however, no studies have assessed the relationship between HRR and frailty intensity. The purpose of this study was to understand the relationship between HRR and the severity of frailty in order to provide a simple and convenient indicator for clinicians to identify frailty risk in a timely manner, for the sake of improving patient quality of life and reducing the risk of complications.

## Materials and Methods

### Ethics Statement

This study passed the ethical batch number (YZUHL20200012) of the School of Nursing, Yangzhou University. Each participant was informed of the purpose of the study and all the procedures involved, and obtained informed consent. This study was undertaken in accordance with the ethical standards of the World Medical Association's Declaration of Helsinki.

### Participants

This was a cross-sectional study that utilized the random sampling method. Elderly patients with CHD in a tertiary hospital in Yangzhou between August 2020 and February 2021 were selected. Subjects were eligible if they met the following inclusion criteria: (1) age ≥ 65 years; (2) meets the diagnostic criteria for coronary atherosclerotic heart disease of the American College of Cardiology; (3) no communication, cognitive, or mental disorders, and able to understand and voluntarily participate in the survey. Patients were excluded if they had any of the following conditions: (1) concomitant infection on admission; (2) in the acute stage of disease, severe cardiopulmonary, renal insufficiency, and terminal disease stage; (3) patients with cancer; (4) patients with New York Heart Association class IV; (5) and patients with missing blood parameters.

### Sociodemographic Characteristics

Sociodemographic characteristics, including age, gender, education years, living situation, monthly income, smoking, alcohol consumption, body mass index (BMI; BMI = height (m)/weight (kg)^2^), number of drugs, comorbidity index (CCI), hypertension, diabetes, heart failure, myocardial infarction, atrial fibrillation, and cerebrovascular diseases were collected using a general questionnaire. Classification was carried out according to the classification method proposed by the micro-quantitative nutritional assessment: 0 = BMI <19; 1 = BMI: 19–21; 2 = BMI: 21–23; 3 = BMI ≥ 23. The number of drugs inquiry was as follows: How many drugs did you take for more than 3 months before hospitalization?

### Peripheral Blood Parameters

Blood parameters at admission were extracted from the hospital electronic information medical record system, including white blood cells (WBC), neutrophils, lymphocytes, monocytes, PLT, red blood cells (RBC), albumin, glucose, total cholesterol, triglyceride, high density lipoprotein cholesterol (HDL-C), low density lipoprotein cholesterol (LDL-C), Hb, and RDW. HRR was calculated using the following formula: HRR = Hb (g/L)/RDW (%).

### Frailty Phenotype Scale

Frailty was evaluated according to the Frailty Phenotype Scale (1), which assesses frailty by measuring five characteristics that include weight loss, slowness, weakness, low physical activity, and exhaustion.

- (1) Weight loss: an unintentional loss of ≥ 4.5 kg or a loss of ≥ 5% of body weight in the past year.
- (2) Slowness: the time required to walk 4.6 m at a normal speed was used as an indicator of slowness. Slow walking speed was defined as ≥ 6 s for a male > 173 cm in height and a female > 159 cm in height or 7 s for a male ≤ 173 cm in height and a female ≤ 159 cm in height.
- (3) Weakness: hydraulic dynamometer was used to measure grip strength as an indicator of weakness. Older adults in a sitting position used the dominant hand to grip an object three times and the researcher recorded the maximum value. Criteria proposed by Fried et al. (1) was used to define weakness.
- (4) Low physical activity: The International Physical Activity Questionnaire was used to assess physical activity (19); males who expended <383 kcal/w and females who expended <270 kcal/w were considered to have low physical activity.
- (5) Exhaustion: poor endurance and energy were assessed using the depression scale, specifically, to check whether the answer to either of these questions is yes: “Last week, I felt like everything I did needed an effort”; “I can't walk forward.” If a positive response was given to either of these questions, the participant was thought to be exhausted.

Frailty scores were 0, 1–2, and ≥ 3, which were divided into non-frail, pre-frail, and frail categories, respectively.

### Data Analysis

SPSS 26.0 (version 26.0, Chicago, IL, USA) was used for data processing and statistical analysis. *P* < 0.05 was considered statistically significant.

Descriptive statistical methods were used to describe the frailty of inpatients. We have introduced the method established by Budczies et al. (20) (at http://molpath.charite.de/cutoff/) to determine the optimal cut-off values for the Hb, the RDW and the HRR.

Continuous variables were represented using mean ± standard deviation, and normal distribution test was performed on the continuous variables. Variables conforming to the normal distribution were compared between groups *via* analysis of variance. Variables not conforming to the normal distribution were compared between groups using a non-parametric test. Classification variables were represented using frequency and composition ratio, and the differences between groups were analyzed using a contingency table.

Kendall's tau-b grade correlation was used to analyze the correlation between peripheral blood Hb, RDW, HRR, and frailty in elderly inpatients with CHD. The analysis was conducted using ordered logistic regression, with frailty as the outcome variable and Hb, RDW, and HRR as independent variables. The independent risk factors for frailty in elderly inpatients with CHD were explored after adjustment for age, BMI, number of drugs, comorbidity index, heart failure, RBC, albumin, total cholesterol, triglyceride, LDL-C and HDL-C.

## Results

### Frailty Status of Elderly Inpatients With CHD

**Figure 1:**
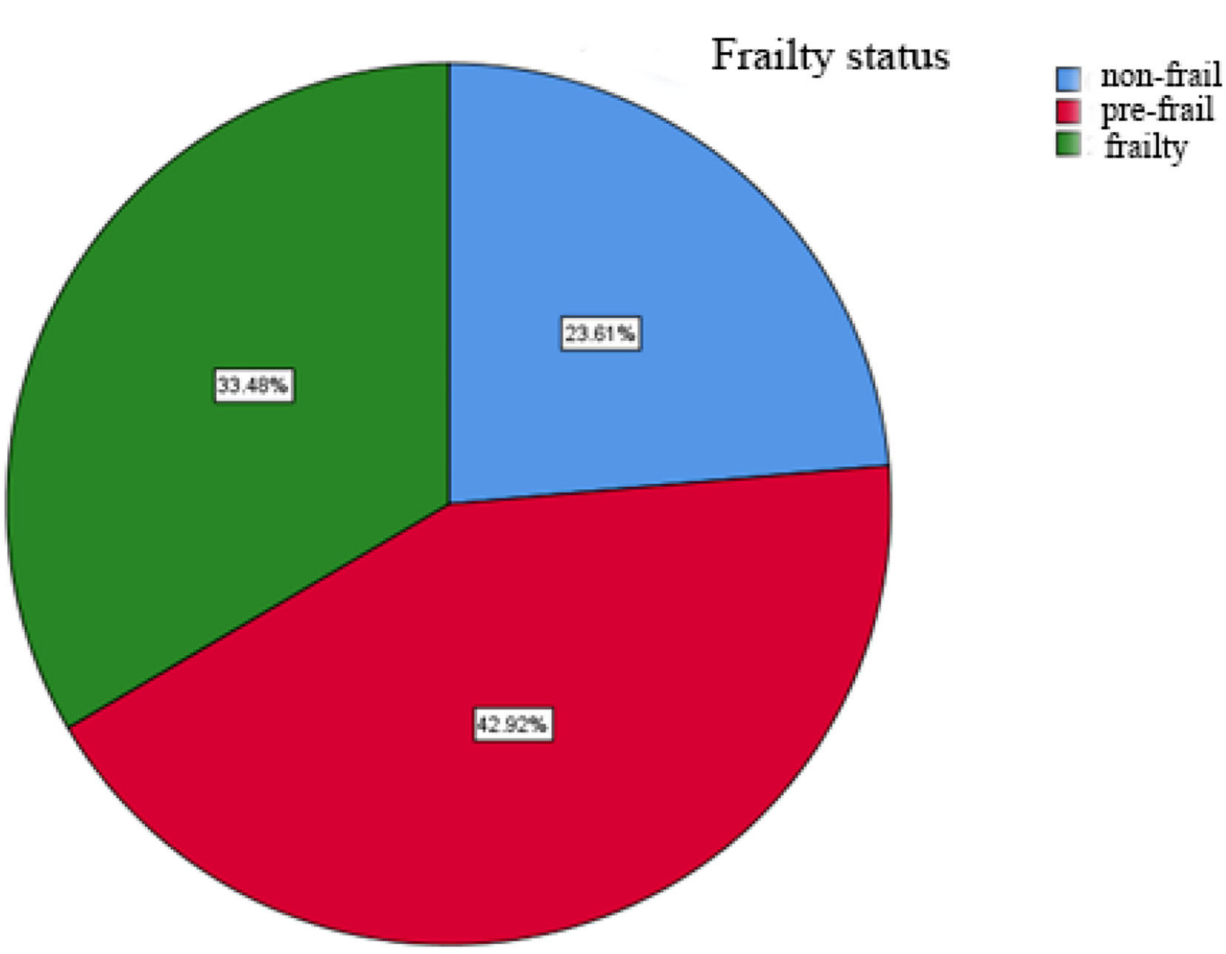
Frailty status of elderly inpatients with CHD.

A total of 245 questionnaires were sent out and 12 invalid questionnaires were eliminated. Thus, 233 valid questionnaires were finally recovered, with an effective recovery rate of 92.1%. The average score on the Fried Scale was 1.88 ± 1.48 points. Among these patients, 23.61% (55) were considered non-frail, 42.92% (100) were pre-frail, and 33.48% (78) were frail (Figure 1).

### Optimal Cut-Off Value for Peripheral Blood Hb, RDW, and HRR for Frailty in Elderly Inpatients With CHD

Patients meeting three or more frailty criteria were included in the frailty group, and the rest were considered to be in the non-frail group. Using frailty as the state variable, the optimal critical values of Hb, RDW, and HRR for frailty were determined using ROC analysis. The AUC for HRR in the frailty patients was 0.652, with the maximum Youden index of 0.268, exceed Hb (AUC = 0.618, the maximum Youden index = 0.205) and RDW (AUC = 0.650, the maximum Youden index = 0.253). All of these results were statistically significant (*P* < 0.05; Table 1).

**Table 1:** Optimal cut-off values for peripheral blood Hb, RDW, and HRR for frailty in elderly inpatients with CHD.

**Items**	**AUC**	**Youden index**	**Sensitivity**	**Specificity**	**Optimal cut-off values**
Hb	0.618	0.205	0.663	0.542	133.50
RDW	0.650	0.253	0.475	0.778	13.35
HRR	0.652	0.268	0.575	0.693	9.76

### Relationship Between Sociodemographic Characteristics and Frailty in Elderly Inpatients With CHD

Univariate analysis of sociodemographic characteristics in elderly patients with CHD showed that age (*P* < 0.001), BMI (*P* = 0.039), number of drugs (*P* < 0.001), CCI (*P* < 0.001), and heart failure (*P* < 0.001) were associated with frailty (Table 2).

**Table 2:** Univariate analysis of sociodemographic characteristics in elderly patients with CHD.

**Items**		**Number of cases (%)**			*****χ^2^**/H***	***P***
		**Non-frail**	**Pre-frail**	**Frailty**		
Age	65~74	44 (38.9)	47 (41.6)	22 (19.5)	41.058^b^	<0.001
	75~84	11 (12.1)	43 (47.3)	37 (40.7)		
	≥85	0 (0.0)	10 (34.5)	65.5 (78)		
Gender	Male	30 (27.3)	47 (42.7)	33 (30.0)	1.941^b^	0.379
	Female	25 (20.3)	53 (43.1)	45 (36.6)		
Education years	0~12	23 (18.4)	59 (47.2)	43 (34.4)	4.316^b^	0.116
	≥13	32 (29.6)	41 (38.0)	35 (32.4)		
Living situation	Alone	5 (19.2)	13 (50.0)	8 (30.8)	0.643^b^	0.725
	Not alone	50 (24.2)	87 (42.0)	70 (33.8)		
Monthly income	<5,000	42 (22.8)	79 (42.9)	63 (34.2)	0.377^b^	0.828
	≥5,000	13 (26.5)	21 (42.9)	15 (33.5)		
Smoking	No	32 (22.4)	(43.4)	49 (34.3)	0.322^b^	0.851
	Yes	23 (25.6)	38 (42.2)	29 (32.2)		
Alcohol consumption	No	28 (19.2)	64 (43.8)	54 (37.0)	4.762^b^	0.092
	Yes	27 (31.0)	36 (41.4)	24 (27.6)		
BMI	<19	0 (0.0)	3 (23.1)	10 (76.9)	13.294^b^	0.039
	19~21	6 (21.4)	13 (46.4)	9 (32.1)		
	21~23	12 (23.5)	25 (49.0)	14 (27.5)		
	>23	37(26.2)	59(41.8)	45(31.9)		
Number of drugs		2.47 ± 2.39^a^	3.87 ± 2.30^a^	4.50 ± 2.95^a^	30.129^c^	<0.001
CCI		3.20 ± 1.01^a^	3.90 ± 1.15^a^	4.62 ± 1.72^a^	38.877^c^	<0.001
Hypertension	No	18 (34.6)	20 (38.5)	14 (26.9)	4.606^b^	0.100
	Yes	37 (20.4)	80 (44.2)	64 (35.4)		
Diabetes	No	43 (24.9)	79 (45.7)	51 (29.5)	4.831^b^	0.089
	Yes	12 (20.0)	21 (35.0)	27 (45.0)		
Heart failure	No	53 (26.8)	89 (44.9)	56 (28.3)	17.472^b^	<0.001
	Yes	2 (5.7)	11 (31.4)	22 (62.9)		
Atrial fibrillation	No	50 (24.4)	88 (42.9)	67 (32.7)	0.766^b^	0.682
	Yes	5 (17.9)	12 (42.9)	11 (39.3)		
Myocardial infarction	No	53 (23.8)	95 (42.6)	75 (33.6)	0.217^b^	0.897
	Yes	2 (20.0)	5 (50.0)	3 (30.0)		
Cerebrovascular diseases	No	49 (23.9)	89 (43.4)	67 (32.7)	0.483^b^	0.786
	Yes	6 (21.4)	11 (39.3)	11 (39.3)		

a *mean ± standard;*

b *chi-square test;*

c *nonparametric tests*.

### Relationship Between Blood Parameters and Frailty in Elderly Patients With CHD

Univariate analysis of blood parameters in elderly patients with CHD showed that RBC (*P* = 0.008), albumin (*P* < 0.001), total cholesterol (*P* = 0.029), triglyceride (*P* = 0.030), HDL-C (*P* = 0.022), LDL-C (*P* = 0.030), Hb (*P* = 0.001), RDW (*P* < 0.001), and HRR (*P* < 0.001) were associated with frailty (Table 3).

**Table 3:** Univariate analysis of blood parameters and frailty in elderly patients with CHD.

**Items**		**Number of cases (%)**			*****χ^2^**/H***	***P***
		**Non-frail**	**Pre-frail**	**Frailty**		
WBC, ×10^9^/L		5.96 ± 1.75^a^	6.30 ± 2.1^a^	6.39 ± 2.57^a^	0.774^c^	0.679
Neutrophil,%		4.19 ± 1.59^a^	4.44 ± 2.06^a^	4.64 ± 2.39^a^	0.969^c^	0.616
Lymphocyte,%		1.33 ± 0.53^a^	1.36 ± 0.57^a^	1.26 ± 0.64^a^	3.810^c^	0.149
Monocytes,%		0.44 ± 0.82^a^	0.38 ± 0.14^a^	0.38 ± 0.16^a^	5.360^c^	0.069
PLT, ×10^9^/L		188.37 ± 66.00^a^	179.17 ± 54.49^a^	170.08 ± 69.24^a^	2.980^c^	0.225
RBC, ×10^12^/L		4.45 ± 0.72^a^	4.28 ± 0.60^a^	4.14 ± 0.71^a^	9.785^c^	0.008
Albumin,g/L		43.06 ± 3.72^a^	41.17 ± 4.00^a^	40.24 ± 3.91^a^	17.760^c^	<0.001
Glucose,mmol/L		7.72 ± 2.63^a^	7.49 ± 3.93^a^	7.75 ± 3.54^a^	1.290^c^	0.525
Total Cholesterol,mmol/L		4.30 ± 0.87^a^	3.92 ± 0.90^a^	3.98 ± 1.17^a^	7.057^c^	0.029
Triglyceride,mmol/L		2.01 ± 1.47^a^	1.57 ± 0.87^a^	1.68 ± 1.14^a^	7.030^c^	0.030
HDL-C,mmol/L		1.11 ± 0.31^a^	1.07 ± 0.29^a^	0.98 ± 0.26^a^	7.678^c^	0.022
LDL-C,mmol/L		2.45 ± 0.72^a^	2.13 ± 0.75^a^	2.21 ± 0.89^a^	7.017^c^	0.030
Hb	<133.50g/L	18 (14.6)	54 (43.9)	51 (41.5)	13.906^b^	0.001
	≥133.50 g/L	37 (33.6)	46 (41.8)	27 (24.5)		
RDW	<3.35%	46 (28.6)	74 (46.0)	41 (25.5)	16.556 ^b^	<0.001
	≥13.35%	9 (12.5)	26 (36.1)	37 (51.4)		
HRR	<9.76	9 (9.7)	39 (41.9)	45 (48.4)	23.034 ^b^	<0.001
	≥9.76	46 (32.9)	61 (43.6)	33 (23.6)		

a *mean ± standard;*

b *chi-square test;*

c *non-parametric tests*.

### Correlation Analysis of Peripheral Blood Hb, RDW, HRR, and Frailty in Elderly Hospitalized Patients With CHD

The correlation between Hb, RDW, HRR, and frailty in peripheral blood of elderly patients with CHD was analyzed using Kendall's tau-b grade. The results indicated that Hb (K = −0.224, *P* < 0.001) and HRR (K = −0.296, *P* < 0.001) were inversely associated with frailty, while RDW (K = 0.248, *P* < 0.001) was positively associated with frailty (Table 4).

**Table 4:** Correlation analysis of peripheral blood Hb, RDW, HRR, and frailty in elderly patients with CHD.

		**Hb**	**RDW**	**HRR**
Frailty	Kendall's tau-b	−0.224	0.248	−0.296
	*P*	<0.001	<0.001	<0.001

### Relationship Between Peripheral Blood Hb, RDW, HRR, and Frailty in Elderly Patients With CHD

Ordered logistic regression analysis was conducted, where the frailty grade (non-frail, pre-frail, and frail) was considered to be the dependent variable, while Hb, RDW, and HRR were the independent variables. Model 1, unadjusted, showed that lower Hb (β = 0.906, *P* < 0.001) and lower HRR (β = 1.236, *P* < 0.001) were associated with frailty risk factors, while lower RDW (β = −1.102, *P* < 0.001) was a protective factor for frailty. In Model 2, after adjusting for age, BMI, number of drugs, CCI and heart failure, lower HRR (β = 0.754, *P* = 0.010) were risk factors for frailty, while lower RDW (β = −0.714, *P* = 0.020) was a protective factor for frailty. In Model 3, after adjusting for age, BMI, number of drugs, CCI, heart failure, RBC, albumin, total cholesterol, triglyceride, LDL-C and HDL-C, lower RDW (β = −0.721, *P* = 0.033) was a protective factor for frailty, while lower HRR (β =1.126, *P* = 0.004) was a risk factor for frailty (Table 5).

**Table 5:** Ordered logistic regression analysis of blood parameters and frailty in elderly patients with CHD.

	**Model 1**			**Model 2**			**Model 3**		
	**β**	***P***	**95% CI**	**β**	***P***	**95% CI**	**β**	***P***	**95% CI**
Lower Hb	0.906	<0.001	0.414~1.399	0.454	0.105	−0.095~1.002	0.728	0.057	−0.022~1.479
Lower RDW	−1.102	<0.001	−1.643~-0.561	−0.714	0.020	−1.315~-0.114	−0.721	0.033	−1.383~-0.059
Lower HRR	1.236	<0.001	0.720~1.752	0.754	0.010	0.178~1.329	1.126	0.004	0.365~1.887

*Model 1, unadjusted; Model 2, adjusted for age, BMI, number of drugs, CCI and heart failure; Model 3, adjusted for age, BMI, number of drugs, comorbidity index, heart failure, RBC, albumin, total cholesterol, triglyceride, LDL-C and HDL-C*.

## Discussion

As the world's elderly population continues to grow, so does the number of frail older people. The number of frail aging individuals is expected to double in the coming decades (21). Whether in clinical nursing or in aging research, frailty is becoming an increasingly important concept. From a pathophysiological perspective, the asthenia syndrome is due to the chronic low-grade and non-infectious state of infectious inflammation (9). In systemic inflammation, inflammatory factors promote the formation of lysophosphatidylcholine, and increased exposure to phosphatidylserine leads to lipid remodeling of erythrocyte membranes, which in turn affects erythrocyte function and longevity. Inflammation also accelerates the clearance of red blood cells by activating macrophages, shortening the lifespan of red blood cells and decreasing hemoglobin (22, 23).

In our study, the Fried scale was used to evaluate 233 elderly inpatients with CHD. It was found that the incidence of frailty was 33.48% (78), which is slightly higher than the prevalence of frailty of 20.8% that was proposed by Hou et al. (24), which may be related to respondent age, condition, and medication. Our study found that pre-frail individuals accounted for about half of the elderly inpatients. Data showed that pre-frail patients were more likely to progress to frailty (25), while certain interventions may delay or even reverse the decline (26). This suggests that clinical workers should pay close attention to pre-frail patients, as their early identification and intervention is more consequential than that of frail patients.

In Table 4, taking frailty as the dependent variable and HRR as the independent variable, Kendall's tau-b grade correlation analysis found that HRR (K = −0.296, *P* < 0.001) was inversely associated with frailty. In Table 5, ordered logistic regression analysis found that in Model 1 without adjustment, Model 2 after adjustment for age, BMI, number of drugs, CCI and heart failure, and Model 3 after adjustment for age, BMI, number of drugs, comorbidity index, heart failure, RBC, albumin, total cholesterol, triglyceride, LDL-C and HDL-C, the lower HRR was an independent risk factor for frailty (*P* < 0.05). At present, specific reasons for the correlation between decreased HRR and frailty remain unclear and are worth exploring further.

A decrease in HRR may be related to a decrease in Hb. That is, patients with a lower HRR are more likely to be in a debilitating state, which may be related to anemia. The prevalence of anemia in patients with acute coronary syndrome in China was found to be 10%~45% (27). In terms of type, the most common anemia associated with CHD was nutritional anemia (47.1%), among which iron deficiency anemia (25.5%), megaloblastic anemia (21.6%) and renal anemia (23.5%) were more common (28). Domestic scholars have found that mild anemia can also aggravate myocardial ischemia, leading to acute coronary syndrome, and found that the incidence of angina is higher than that of non-anemia group. When coronary artery occlusion is incomplete, anemia will break the micro-balance between myocardial oxygen supply and myocardial oxygen consumption, and then lead to the occurrence of myocardial ischemia (29).

A study on the correlation between anemia and frailty in the elderly from Spain (30) found that the probability of anemia occurrence in the elderly was 19.6%, while the prevalence of anemia in frail individuals was 29.6%, significantly higher than that in the pre-frail (16.6%) and non-frail (6%). In the fully adjusted regression model, anemia was associated with frailty (OR = 1.95, 95%CI: 1.02–3.73, *P* < 0.05). Consistent with the results of this study, Hb (K = −0.224, *P* < 0.001) was negatively correlated with frailty in the present investigation. Ordered logistic regression analysis found that in Models 1, lower Hb was an independent risk factor for frailty (*P* < 0.001), although no statistical significance was observed in Model 3 (*P* = 0.105)and Model 4 (*P* = 0.057).

Anemia reduces the ability of RBCs to carry oxygen, causing tissue to lack oxygen and leading to a number of adverse outcomes, including reduced aerobic capacity, decreased muscle strength, cognitive impairment, and increased fatigue, which can also increase the risk of frailty in older people (31). Röhrig (32) found that underlying diseases and inflammation can lead to chronic inflammatory anemia, the most common type of anemia in the elderly. Increased RBC adherence to the endothelium during inflammation is most likely due to the increased expression of endothelial adhesion molecules and phosphatidylserine on the erythrocyte membrane, as well as decreased capillary blood flow (33). Therefore, primary health care providers need to pay attention to anemia, determine its causes, and execute timely correction in order to prevent the occurrence of frailty.

A decrease in HRR is not only related to a decrease in Hb, but also to an increase in RDW. The present study found that RDW (K = 0.248, *P* < 0.001) was positively correlated with frailty, which was confirmed in the regression analysis. In addition, RDW was an independent risk factor for frailty in elderly patients with CHD. This is consistent with the results by Hou et al. (10). Ming et al. (12) analyzed 2,932 elderly community-dwelling adults and found that elevated RBCs were independently associated with a higher risk of frailty. Frailty is associated with an altered inflammatory status. Inflammation can increase erythrocyte clearance, inhibit erythropoietin, reduce iron utilization, and increase RDW (33). Some studies have shown that RDW is also significantly associated with other inflammatory cytokines, such as serum malondialdehyde levels, tumor necrosis factor-α, interleukin-6, and interleukin-10 (34, 35).

Both Hb and RDW are important components of HRR, and the relationship between lower Hb, higher RDW, and frailty in elderly CHD patients has been confirmed. However, considering that both Hb and RDW are susceptible to diseases other than CHD, HRR may be a more reliable parameter. The predictive analysis showed that AUC, Youden index, sensitivity, and specificity of HRR in senile frailty patients were 0.652, 0.268, 0.575, and 0.693, respectively. AUC and Youden index were higher than Hb and RDW.

There are some limitations in the present study. First, the study was only conducted in a tertiary hospital and cannot be considered comprehensive. It is hoped that there will be multi-center, large sample trials in the future. Second, only the blood parameters upon admission were obtained in the study. If the blood parameters of interest were measured, the relationship between them and frailty might be better described. Third, due to the limited knowledge of drugs in the elderly, the collection of names of drugs taken before admission was largely missing, and the drug type was not analyzed to exclude its influence on frailty. In addition, this was a cross-sectional study that can only represent an association between blood parameters and frailty, but not a cause-and-effect relationship. We will continue a further follow-up study of patients' details.

## Conclusion

In conclusion, lower HRR is associated with increased frailty risk and has a certain predictive value in elderly hospitalized patients with CHD. As a simple, effective, and economical blood parameter, HRR should be carefully considered by clinicians in order to identify the risk of frailty in a timely manner, and to monitor the effectiveness of frailty interventions, delay the progression of frailty, prevent the occurrence of disability, reduce adverse outcomes and medical costs, and improve quality of life.

## Data Availability Statement

The original contributions presented in the study are included in the article, further inquiries can be directed to the corresponding author.

## Ethics Statement

The studies involving human participants were reviewed and approved by the Ethics Review Committee of the School of Nursing, Yangzhou University (ethical batch number: YZUHL20200012). The patients/participants provided their written informed consent to participate in this study.

## Author Contributions

YL conceived the study. JQ, TZ, MX, HS, YS, YC, LT, LQ, JY, and RY collected, verified, and analyzed the data. JQ drafted the manuscript. All authors provided critical revision of the manuscript for important intellectual content.

## Conflict of Interest

The authors declare that the research was conducted in the absence of any commercial or financial relationships that could be construed as a potential conflict of interest.

## Publisher's Note

All claims expressed in this article are solely those of the authors and do not necessarily represent those of their affiliated organizations, or those of the publisher, the editors and the reviewers. Any product that may be evaluated in this article, or claim that may be made by its manufacturer, is not guaranteed or endorsed by the publisher.

## References

[1] Fried L Tangen C Walston J Newman A Hirsch C Gottdiener J. Frailty in older adults: evidence for a phenotype. J Gerontol A Biol Sci Med Sci. (2001) 56:M146–56. 10.1093/gerona/56.3.M146 11253156

[2] Chehrehgosha M Fadaye VR Alizadeh KM Sharifi F Aminalroaya R Vahabi Z. Role of frailty in prediction of hospitalized older adult patients outcomes: a prospective study. Turk J Med Sci. (2021) 33843174 10.3906/sag-2012-332 PMC8742491

[3] Gao K Li B Yang L Zhou D Ding K Yan J. Cardiometabolic diseases, frailty, and healthcare utilization and expenditure in community-dwelling Chinese older adults. Sci Rep. (2021) 11:7776. 10.1038/s41598-021-87444-z 33833338 PMC8032763

[4] Kawada T. Frailty and all-cause mortality in older adults: a risk assessment. J Am Med Dir Assoc. (2021) 22:1774. 10.1016/j.jamda.2021.03.006 33839090

[5] Damluji AA Chung SE Xue QL Hasan RK Walston JD Forman DE. Physical frailty phenotype and the development of geriatric syndromes in older adults with coronary heart disease. Am J Med. (2021) 134:662–71.e1. 10.1016/j.amjmed.2020.09.057 33242482 PMC8107119

[6] Yoshioka N Takagi K Morita Y Yoshida R Nagai H Kanzaki Y. Impact of the clinical frailty scale on mid-term mortality in patients with ST-elevated myocardial infarction. Int J Cardiol Heart Vasc. (2019) 22:192–8. 10.1016/j.ijcha.2019.02.014 30963094 PMC6437299

[7] Rodriguez-Mañas L Fried LP. Frailty in the clinical scenario. Lancet. (2015) 385:e7–9. 10.1016/S0140-6736(14)61595-6 25468154

[8] Kojima G Taniguchi Y Iliffe S Jivraj S Walters K. Transitions between frailty states among community-dwelling older people: a systematic review and meta-analysis. Ageing Res Rev. (2019) 50:81–8. 10.1016/j.arr.2019.01.010 30659942

[9] Bodolea C Hiriscau EI Buzdugan EC Grosu AI Stoicescu L Vesa S. The association between peripheral blood cells and the frailty syndrome in patients with cardiovascular diseases. Endocr Metab Immune Disord Drug Targets. (2020) 20:1419–33. 10.2174/1871530320666200813135905 32787768 PMC8226153

[10] Hou P Xue H Mao X Li Y Wu L Liu Y. Inflammation markers are associated with frailty in elderly patients with coronary heart disease. Aging. (2018) 10:2636–45. 10.18632/aging.101575 30325739 PMC6224228

[11] Mailliez A Guilbaud A Puisieux F Dauchet L Boulanger É. Circulating biomarkers characterizing physical frailty: CRP, hemoglobin, albumin, 25OHD and free testosterone as best biomarkers. Results of a meta-analysis. Exp Gerontol. (2020) 139:111014. 10.1016/j.exger.2020.111014 32599147

[12] Li C-M Chao C-T Chen S-I Han D-S Huang K-C. Elevated red cell distribution width is independently associated with a higher frailty risk among 2,932 community-dwelling older adults. Front Med. (2020) 7:470. 10.3389/fmed.2020.00470 32984367 PMC7477345

[13] Nishijima T Deal A Williams G Guerard E Nyrop K Muss H. Frailty and inflammatory markers in older adults with cancer. Aging. (2017) 9:650–64. 10.18632/aging.101162 28273043 PMC5391224

[14] Cheong C Nyunt M Gao Q Gwee X Choo R Yap K. Risk factors of progression to frailty: findings from the singapore longitudinal ageing study. J Nutr Health Aging. (2020) 24:98–106. 10.1007/s12603-019-1277-8 31886815 PMC12879086

[15] Cift A Yucel MO. Comparison of inflammatory markers between brucella and non-brucella epididymo-orchitis. Int Braz J Urol. (2018) 44:771–8. 10.1590/s1677-5538.ibju.2018.0004.0 29697933 PMC6092658

[16] Zhang ZH Xu X Ni HY Deng HS. Red cell distribution width is associated with hospital mortality in unselected critically ill patients. J Thorac Dis. (2013) 5:730–6. 10.3978/j.issn.2072-1439.2013.11.14 24409348 PMC3886701

[17] Horta-Baas G Romero-Figueroa MD. Clinical utility of red blood cell distribution width in inflammatory and non-inflammatory joint diseases. Int J Rheum Dis. (2019) 22:47–54. 10.1111/1756-185X.13332 30168259

[18] Sun P Zhang F Chen C Bi XW Yang H An X. The ratio of hemoglobin to red cell distribution width as a novel prognostic parameter in esophageal squamous cell carcinoma: a retrospective study from southern China. Oncotarget. (2016) 7:42650–60. 10.18632/oncotarget.9516 27223088 PMC5173164

[19] Liou YM Jwo CJC Yao KG Chiang L-C Huang L-H. Selection of appropriate Chinese terms to represent intensity and types of physical activity terms for use in the Taiwan version of IPAQ. J Nurs Res. (2008) 16:252–63. 10.1097/01.JNR.0000387313.20386.0a 19061172

[20] Budczies J Klauschen F Sinn B Gyorffy B Schmitt W Darb-Esfahani S. Cutoff finder: a comprehensive and straightforward web application enabling rapid biomarker cutoff optimization. PLoS ONE. (2012) 7:e51862. 10.1371/journal.pone.0051862 23251644 PMC3522617

[21] Etman A Burdorf A Van der Cammen T Van Lenthe F. Socio-demographic determinants of worsening in frailty among community-dwelling older people in 11 European countries. J Epidemiol Community Health. (2012) 66:1116–21. 10.1136/jech-2011-200027 22544921

[22] Dinkla S van Eijk L Fuchs B Schiller J Joosten I Brock R. Inflammation-associated changes in lipid composition and the organization of the erythrocyte membrane. BBA Clin. (2016) 5:186–92. 10.1016/j.bbacli.2016.03.007 27200268 PMC4864322

[23] Palaka E Grandy S van Haalen H McEwan P Darlington O. The impact of CKD anaemia on patients: incidence, risk factors, and clinical outcomes-a systematic literature review. Int J Nephrol. (2020) 2020:7692376. 10.1155/2020/7692376 32665863 PMC7349626

[24] Hou P Xue H Li Y Mao X Sun K Xue L. Performance of the FRAIL scale in screening frailty among elderly patients with coronary heart disease. Chin Gen Pract. (2019) 22:1052–6. 10.12114/j.issn.1007-9572.2018.00.14925

[25] Liu ZY Wei YZ Wei LQ Jiang XY Wang XF Shi Y. Frailty transitions and types of death in Chinese older adults: a population-based cohort study. Clin Interv Aging. (2018) 13:947–56. 10.2147/CIA.S157089 29805253 PMC5960243

[26] Courel-Ibáñez J Vetrovsky T Dadova K Pallarés JG Steffl M. Health benefits of β-hydroxy-β-methylbutyrate (HMB) supplementation in addition to physical exercise in older adults: a systematic review with meta-analysis. Nutrients. (2019) 11:2082. 10.3390/nu11092082 31484462 PMC6769498

[27] Weiyi F Hongyu S. Acute coronary syndrome and anemia. J Cardiopulm Vasc Dis. (2007) 26:182–184. 10.3969/j.issn.1007-5062.2007.03.022

[28] Kalra PR Greenlaw N Ferrari R Ford I Tardif J-C Tendera M. Hemoglobin and change in hemoglobin status predict mortality, cardiovascular events, and bleeding in stable coronary artery disease. Am J Med. (2017) 130:720–30. 10.1016/j.amjmed.2017.01.002 28109968

[29] Shanshan L Fonarow GC Mukamal KJ Li L Schulte PJ Smith EE. Sex and race/ethnicity-related disparities in care and outcomes after hospitalization for coronary artery disease among older adults. Circ Cardiovasc Qual Outcomes. (2016) 9:S36–44. 10.1161/CIRCOUTCOMES.115.002621 26908858

[30] Esquinas-Requena J García-Nogueras I Hernández-Zegarra P Atienzar-Núñez P Sánchez-Jurado P Abizanda P. Anemia and frailty in older adults from Spain. FRADEA Study. Rev Esp Geriatr Gerontol. (2021) 56:129–35. 10.1016/j.regg.2021.01.010 33771359

[31] Zhao Y. Value of procalcitonin erythrocyte distribution width in the evaluation of septicemia and prognosis of premature infants (Master's Thesis). Anhui Medical University, Anhui, China (2020).

[32] Röhrig G. Anemia in the frail, elderly patient. Clin Interv Aging. (2016) 11:319–26. 10.2147/CIA.S90727 27051279 PMC4803240

[33] Straat M van Bruggen R de Korte D Juffermans N. Red blood cell clearance in inflammation. Transfus Med Hemother. (2012) 39:353–61. 10.1159/000342229 23801928 PMC3678279

[34] He Y Liu C Zeng Z Ye W Lin J Ou Q. Red blood cell distribution width: a potential laboratory parameter for monitoring inflammation in rheumatoid arthritis. Clin Rheumatol. (2018) 37:161–7. 10.1007/s10067-017-3871-7 29101675

[35] Lorente L Martín M Abreu-González P Solé-Violán J Ferreres J Labarta L. Red blood cell distribution width during the first week is associated with severity and mortality in septic patients. PLoS ONE. (2014) 9:e105436. 10.1371/journal.pone.0105436 25153089 PMC4143268

